# Stiffer Matrix Accelerates Migration of Hepatocellular Carcinoma Cells through Enhanced Aerobic Glycolysis Via the MAPK-YAP Signaling

**DOI:** 10.3390/cancers12020490

**Published:** 2020-02-19

**Authors:** Qiu-Ping Liu, Qing Luo, Bin Deng, Yang Ju, Guan-Bin Song

**Affiliations:** 1 Key Laboratory of Biorheological Science and Technology, Ministry of Education, College of Bioengineering, Chongqing University, Chongqing 400030, China; liuqp@cqu.edu.cn (Q.-P.L.); qing.luo@cqu.edu.cn (Q.L.); dengbin2018@cqu.edu.cn (B.D.); 2 Department of Mechanical Science and Engineering, Nagoya University, Nagoya 464-8603, Japan; ju@mech.nagoya-u.ac.jp

**Keywords:** hepatocellular carcinoma, ECM stiffness, migration, aerobic glycolysis, mechanotransduction

## Abstract

Increased extracellular matrix (ECM) stiffness and metabolic reprogramming of cancer cells are two fundamental mediators of tumor progression, including hepatocellular carcinoma (HCC). Yet, the correlation between ECM stiffness and excessive aerobic glycolysis in promoting the development of HCC remains unknown. Here, we demonstrated that stiffer ECM promotes HCC cell migration depending on their accelerated aerobic glycolysis. Our results also indicated that stiffer ECM-induced YAP activation plays a major role in promoting aerobic glycolysis of HCC cells. Moreover, we showed that JNK and p38 MAPK signaling are critical for mediating YAP activation in HCC cells. Together, our findings established that the MAPK-YAP signaling cascade that act as a mechanotransduction pathway is essential for promoting HCC cell aerobic glycolysis and migration in response to ECM stiffness.

## 1. Introduction

Tumors generate physical forces during tumorigenesis, including solid stress, interstitial fluid pressure and extracellular matrix (ECM) stiffness [1]. These physical forces are continuously applied to cancer cells within tumor tissues and dynamically modify their behaviors [2,3]. In recent years, tumor mechanics, tissue stiffness in particular, has emerged as an important factor that correlates with cancer progression and aggression [4]. Human breast cancer invasion and aggression were shown to correlate with ECM stiffening [5]. Hepatocellular carcinoma (HCC) as one of the commonest human solid tumors also has the feature of increased stiffness of the liver tissue, accompanied with abnormal angiogenesis. Studies reported that ECM stiffness regulated cytokines and integrins that involved in modulating HCC angiogenesis and the advancement of HCC [6]. For example, Pang et al. reported that ECM stiffness modulated the TGF-β1 activation and thus promoted the advancement of HCC [7]; Zhao et al. and Dong et al. suggested that ECM stiffness acted as initiators to modulate the development of HCC [8] and HCC angiogenesis via regulated the expression of integrin β1 [9]. Furthermore, studies have suggested that ECM stiffness plays an important role in modulating HCC cell behaviors, including proliferation, chemotherapeutic response, and dormancy [10]. There are increasing evidence suggest that mechanical signals in cellular microenvironment are important regulators of cell behaviors.

Cancer occurs gradually after the accumulation of genetic and epigenetic changes in normal cells, accompanied by changes in energy metabolism. Increased aerobic glycolysis (Warburg effect) is the main source of energy supply in cancer cells. Importantly, glycolysis inhibition induces cancer cell death and results in sensitized cancer cells to chemotherapeutics and reduced tumorigenicity [11,12,13], suggesting that the aerobic glycolytic is central to tumor growth and survival [14]. Like many other cancer cells, accelerated aerobic glycolysis and consequent lactate production have been observed in HCC cells [15,16,17], which are associated with poor outcome in human HCC. In addition, study indicated that inhibition of aerobic glycolysis significantly impaired both the growth and migratory potential of HCC cells [18]. Thus, a better understanding of the mechanism of cellular metabolic changes in the occurrence and development of HCC could be of great significance for the development of new therapeutics for HCC.

Tumor niche ECM stiffening and tumor cell metabolic reprogramming are two fundamental mediators of tumor progression [19]. Emerging evidence indicates that metabolism change occurs downstream of mechanotransduction signaling pathways that are activated by mechanical stimuli [20,21]. Little is known; however, on the causal relationships between cell aerobic glycolytic metabolism, mechanical forces-induced fate and the biomechanical signals transduction during this process.

The transcription factor Yes-associated protein (YAP) has been identified as universal mechanotransducer [22], which is deregulated in diverse cancers, including HCC [23]. As Zhang et al. recently reviewed “increased YAP/TAZ levels and activities could contribute liver tumorigenesis” [23]. Moreover, recent studies showed that YAP contribute to cancer metabolic reprogramming [21,24], including glycolysis. As studies reported, the activation of YAP can promote glucose uptake and the rate of glycolysis in tumor cells [25,26,27]. However, few studies have reported on the relationship between the two roles of YAP.

Many types of upstream signaling related to carcinogenesis have been found to activate YAP. For example, PI3K/Akt, Wnt, and Notch signaling, have been shown to regulate YAP/TAZ activity in liver cancer development [28,29]. The mitogen-activated protein kinase (MAPK) family participates in a variety of cellular functions. In addition, evidence suggested that mechanical force loads can stimulate the activation of one or more members of the MAPK family to regulate cell behaviors [30,31,32]. Therefore, we suppose that the mechanical properties of the ECM also regulate YAP by influencing MAPK signaling. In the present study, we aim to explore the relationship between HCC cell migration and glycolysis regulated by the mechanical feature of ECM, as well as the role of the MAPK and YAP signaling pathway.

## 2. Results

### 2.1. Matrix Stiffness Affects the Migration of HCC Cells

**Figure 1:**
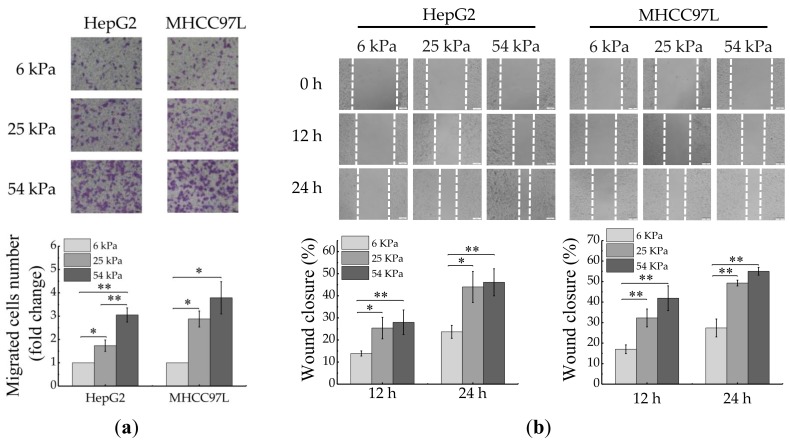
Migration of HCC cells is regulated by ECM stiffness. (**a**,**b**) Transwell assay analysis (**a**) and wound scratch assay analysis (**b**) of HepG2 and MHCC97L cells migration after cultured on different stiffness of hydrogel for 48 h; quantitative analysis is shown below. (Scale bar: 100 µm; *n* = 3, * *p* < 0.05, ** *p* < 0.01).

To investigate the effect of ECM stiffness on the migration of HCC cells, we prepared polyacrylamide (PA) hydrogels with three kinds of stiffness (6, 25 and 54 kPa, matching the physiological elasticities of normal liver, cirrhosis and HCC tissues [33,34,35]). After cultured for 48 h, the migration ability of HCC cells was measured by using wound scratch assay and transwell assay. The results showed that the migration ability of HepG2 and MHCC97L cells all significantly increased with the increase of hydrogel stiffness (Figure 1a,b).

### 2.2. Stiffer Matrix Promotes the Migration of HCC Cells via Upregulating Aerobic Glycolysis

**Figure 2:**
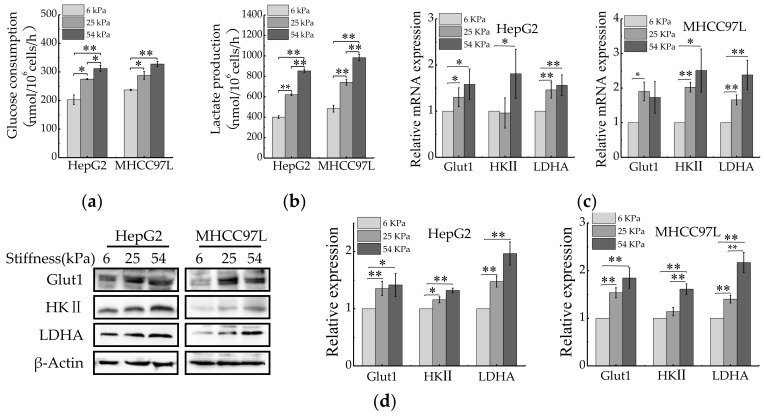
Aerobic glycolysis of HCC cells is regulated by ECM stiffness. (**a**,**b**) Measurement of glucose consumption (**a**) and lactate production (**b**) of HepG2 and MHCC97L cells cultured on different stiffness of hydrogel for 48 h. (**c**) qRT-PCR analysis of indicated genes mRNA levels in HCC cells. (**d**) Western blot analysis of indicated protein levels in HCC cells. (*n* = 3, * *p* < 0.05, ** *p* < 0.01).

Aerobic glycolysis is a metabolic hallmark of most cancer cells, including HCC cells, characterized by excessive consumption of glucose and huge production of lactate, whether or not oxygen is present [36]. We were curious about whether ECM stiffness would also regulate aerobic glycolysis of HCC cells. After being cultured for 48 h, the levels of glucose consumption and lactate production of HCC cells were measured. Compared with 6 kPa, HCC cells cultured on stiffer matrix resulted in increasing glucose consumption and lactate production (Figure 2a,b). As cancer cells accelerated glycolysis generally by preferential expression of glucose transporters (e.g., Glut1) [18] and key glycolytic enzymes (e.g., HK II and LDHA) [37,38]. We therefore explored whether ECM stiffness regulates expression of these glycolysis-associated enzymes. As expected, significant up-regulation of Glut1, HK II and LADH were observed in HCC cell lines cultured on the stiffer matrix (Figure 2c,d). Collectively, these results suggested that ECM stiffness might be a regulator of aerobic glycolysis in human cancers.

**Figure 3:**
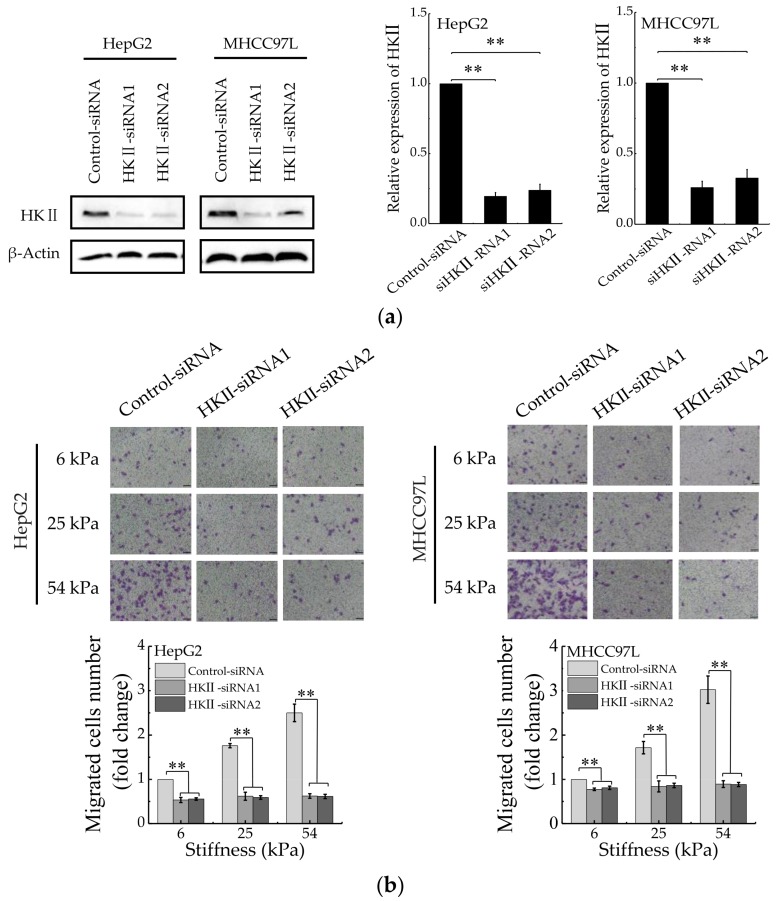

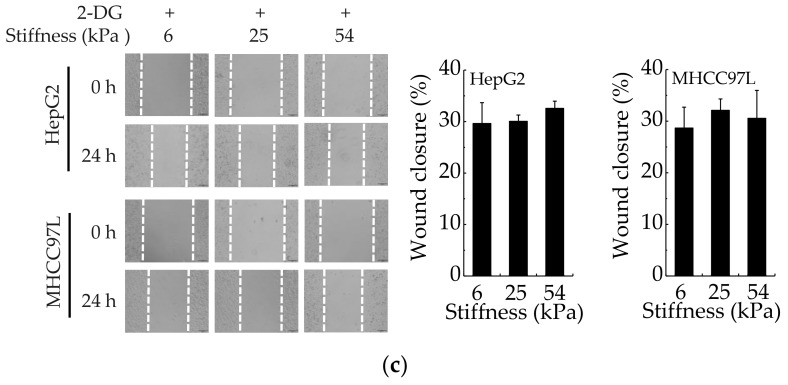
Aerobic glycolysis is responsible for stiffer ECM-mediated migration. (**a**) Western blot analysis showed the protein expression of HKII in HepG2 and MHCC97L after knockdown of HKII (*n* = 3, ** *p* < 0.01 versus control-siRNA group). (**b**) Transwell analysis of HKII-knockdown HepG2 and MHCC97L cells migration (Scale bar: 100 µm; *n* = 3, ** *p* < 0.01). (**c**) Wound scratch assay analysis of HepG2 and MHCC97L cells after treatment with 2-DG (20 mM) (Scale bar: 100 µm; *n* = 3).

To understand the correlation between HCC cell migration and glycolysis regulated by matrix stiffness, the migration ability of HCC cells was detected after HKII knockdown with specific siRNAs (Figure 3a). We found that compared with control silencing group, the migration of HCC cells in HKII-knockdown groups decreased significantly (Figure 3b). Moreover, after HKII knockdown, the migration of HCC cells has no significant difference when cultured on different stiffness of hydrogels. In addition, the results are consistent after inhibiting glycolysis of HCC cells with 2-Deoxy-D-glucose (2-DG) (Figure 3c). Taken together, these results argued that stiffer matrix promotes the migration of HCC cells via upregulating their aerobic glycolysis.

### 2.3. ECM Stiffness Regulates YAP Activation

**Figure 4:**
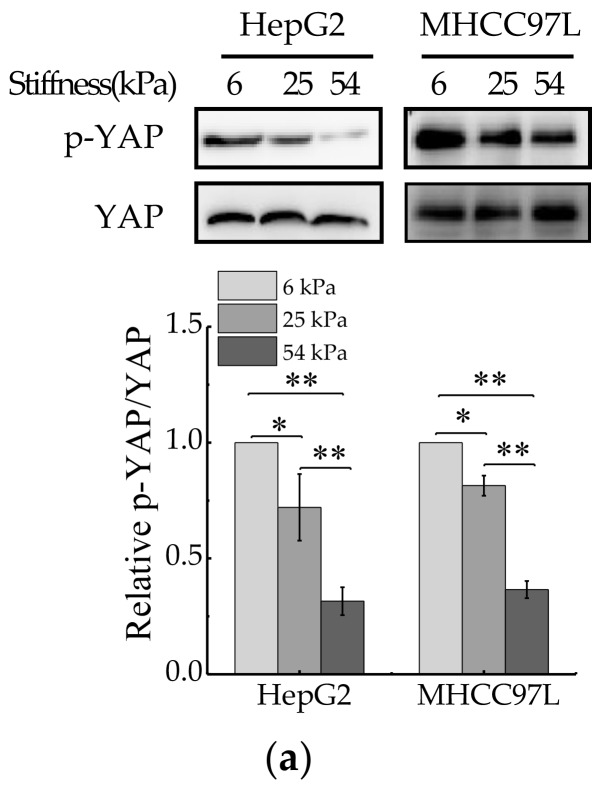

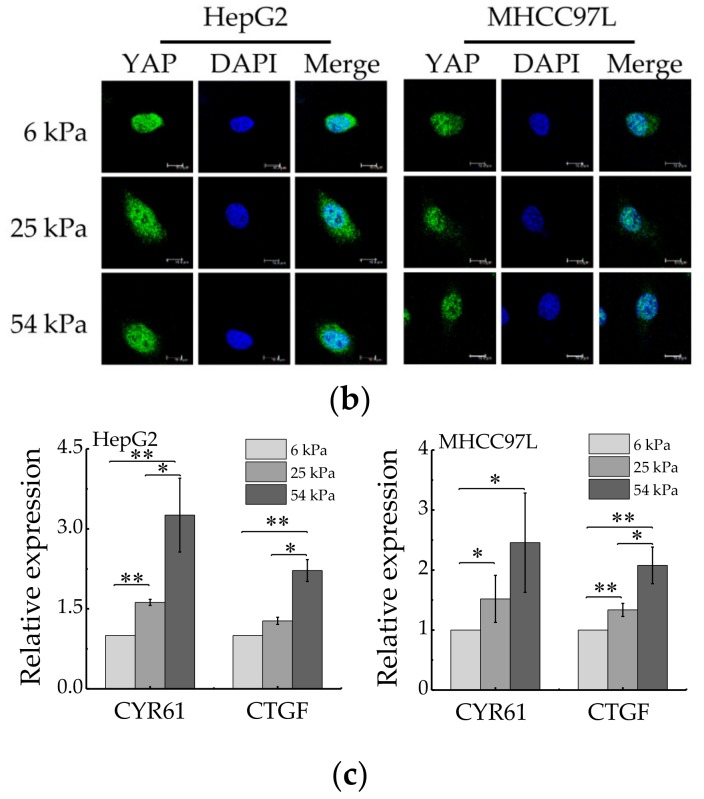
YAP activity is regulated by ECM stiffness. (**a**) Western blot analysis of total YAP and p-YAP protein levels in HepG2 and MHCC97L cells cultured on different stiffness of hydrogel for 48 h (*n* = 3, * *p* < 0.05, ** *p* < 0.01). (**b**) qRT-PCR analysis of indicated genes mRNA levels in HepG2 and MHCC97L cells (*n* = 3, * *p* < 0.05, ** *p* < 0.01). (**c**) Confocal immunofluorescence images of YAP in HepG2 and MHCC97L cells cultured on different stiffness of hydrogel for 48 h (Scale bar: 25 µm).

YAP is a sensor of mechanical features of the cell microenvironment. To test whether YAP is regulated by ECM stiffness, we monitored YAP activity in human HCC cells grown on Collagen Type I (COL1)-coated PA hydrogels of varying stiffness. For this, we primly performed western blot to measure expression of total YAP and phosphorylated YAP (p-YAP). The results showed that the ratio of p-YAP/total YAP decreased with the increase of hydrogel stiffness (Figure 4a). Then, immunofluorescent assay was also conducted to assay endogenous YAP subcellular localization, as the phosphorylated form of YAP localizes in the cytoplasm and the dephosphorylated form of YAP localizes in the nucleus, where it interacts with other transcription factors [39]. The result showed that YAP localized in cytoplasm and nucleus in HCC cells cultured on soft hydrogel and the amount of YAP localized in the nucleus increased with the increase of matrix stiffness (Figure 4b). Furthermore, the expression of YAP target genes, CTGF and CYR61, were assayed by quantitative real-time PCR (qRT-PCR). The results showed that the mRNA levels of CTGF and CYR61 all increased with the increase of hydrogel stiffness (Figure 4c), which indicated the transcriptional activity of YAP increased in HCC cells cultured on stiffer hydrogels. Collectively, these data indicated that YAP activity and subcellular localization are regulated by ECM stiffness.

### 2.4. YAP is a Key Mediator in Regulating Stiffer ECM-Induced Excessive Aerobic Glycolysis

**Figure 5:**
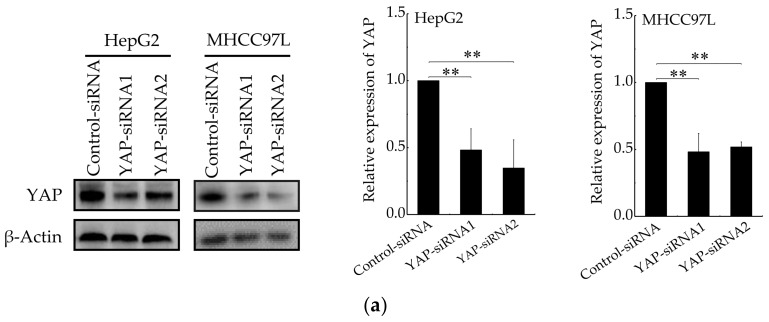

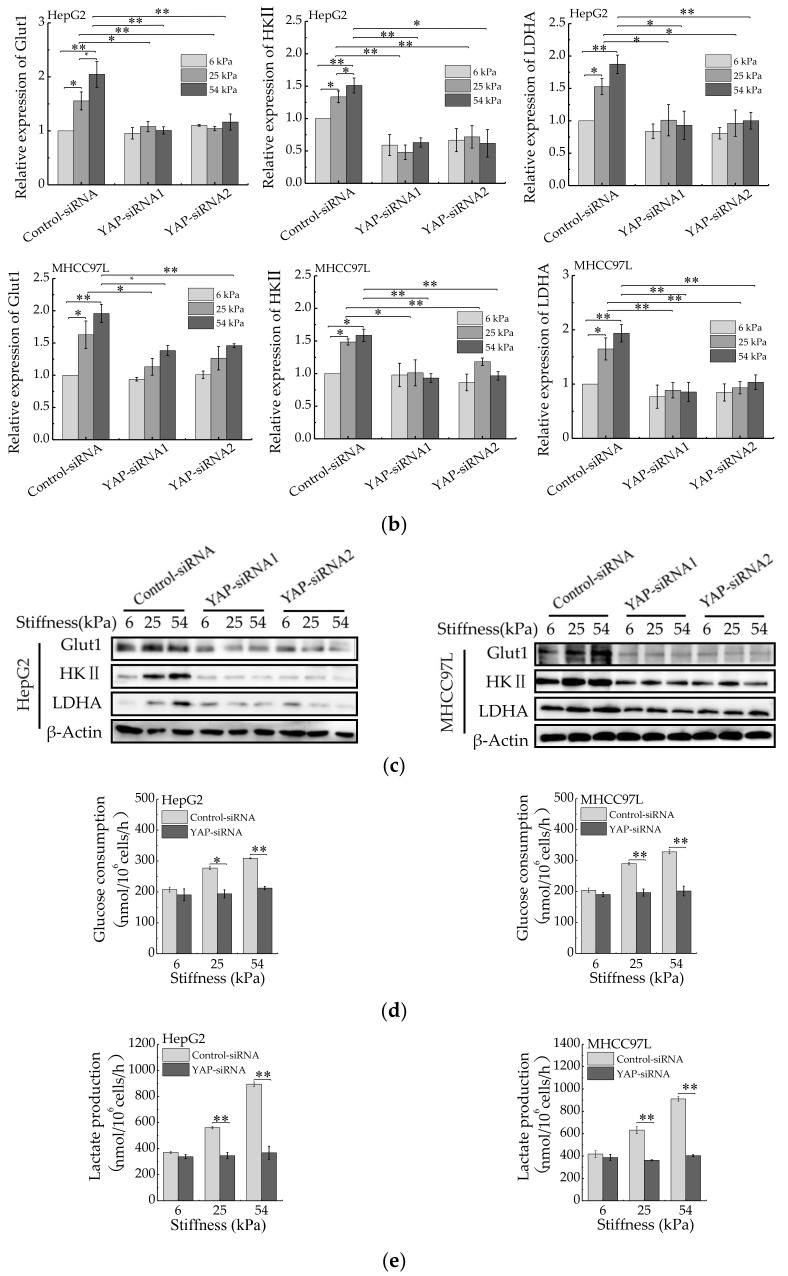
YAP is responsible for ECM stiffness-mediated aerobic glycolysis. (**a**) Western blot analysis of YAP expression in HepG2 and MHCC97L cells after YAP knockdown (*n* = 3, ** *p* < 0.01 versus control-siRNA group). (**b**) qRT-PCR analysis of indicated genes mRNA levels in YAP-knockdown HepG2 and MHCC97L cells. (**c**) Western blot analysis of indicated proteins in YAP-knockdown HepG2 and MHCC97L cells. (**d**,**e**) Measurement of glucose consumption (**d**) and lactate production (**e**) in HepG2 and MHCC97L cells after YAP knockdown (*n* = 3, * *p* < 0.05, ** *p* < 0.01).

We next determined whether YAP is responsible for aerobic glycolysis of HCC cells. Two specific siRNAs were used to knockdown YAP (Figure 5a). Then, qRT-PCR and western blot were conducted to detect the expression of Glut1, HKII and LADH. As expected, the expression of Glut1, HKII and LADH (Figure 5b,c) were reduced in YAP-knockdown HepG2 and MHCC97L cells cultured on stiffer hydrogels. Consistently, the knockdown of YAP also decreased glucose consumption and lactate production (Figure 5d,e) in HCC cells cultured on stiffer hydrogels. Moreover, the expression of glycolysis-related enzymes, glucose consumption and lactate production have no striking difference in YAP-knockdown HCC cells cultured on different stiffness of hydrogels. These findings suggested that YAP is responsible for stiffer ECM-induced accelerating aerobic glycolysis.

### 2.5. JNK and p38 MAPK Signaling Regulate Stiffer ECM-Induced YAP Activation and HCC Cells Migration

**Figure 6:**
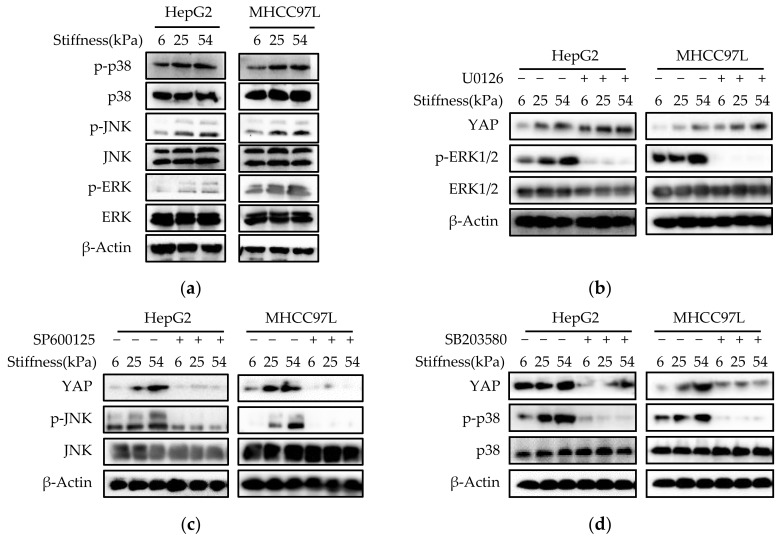

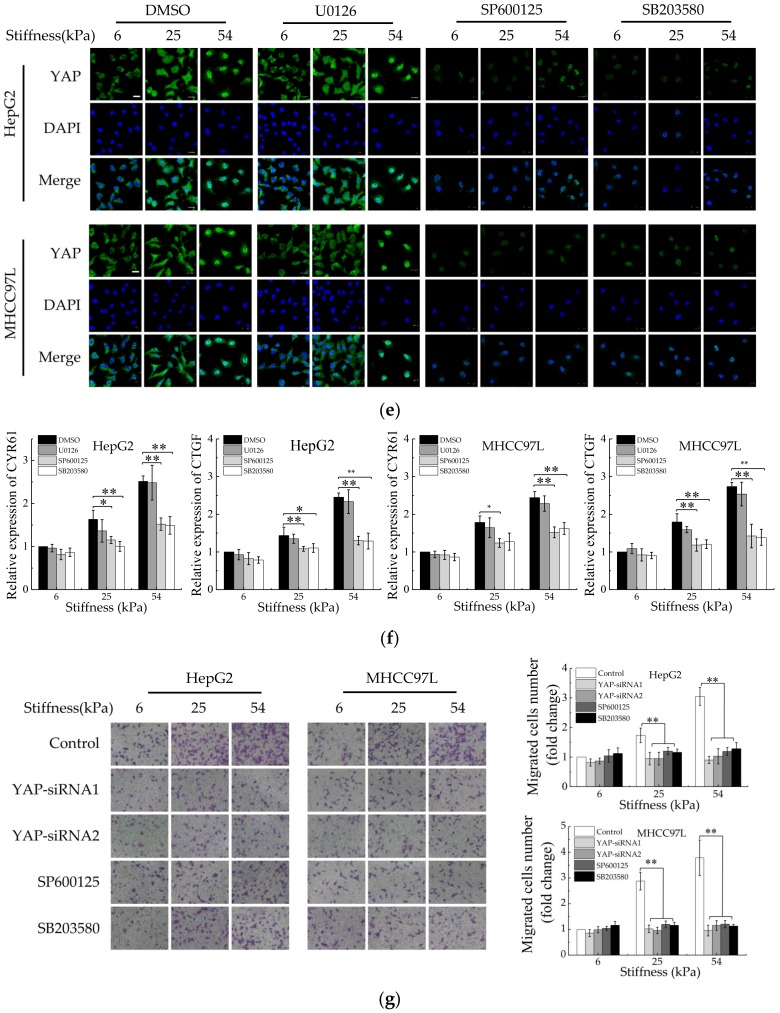
JNK and p38 MAPK signaling regulate stiffer ECM-induced YAP activation. (**a**) Western blot analysis of the indicated proteins in HepG2 and MHCC97L cells cultured on different stiffness of hydrogels for 48 h. (**b,c,d**) Western blot analysis of the indicated proteins in HepG2 and MHCC97L cells treated with U0126 (10 μM), SP600125 (20 μM), SP600125 (10 μM) or vehicle. (**e**) Confocal immunofluorescence images of YAP in HepG2 and MHCC97L cells after treated with U0126, SP600125, SP600125 or vehicle (Scale bar: 25 µm). (**f**) qRT-PCR analysis of indicated genes mRNA levels in HepG2 and MHCC97L cells after treated with U0126, SP600125, SP600125 or vehicle (*n* = 3, * *p* < 0.05, ** *p* < 0.01). (**g**) Transwell assay analysis of HepG2 and MHCC97L cells migration after indicated treatment (*n* = 3, * *p* < 0.05, ** *p* < 0.01).

Having established that YAP plays a key role in mediating stiffer ECM-induced migration of HCC cells; we wanted to explore which signals regulate stiffer ECM-induced YAP activation and nuclear localization in HCC cells. Studies have shown that increased mechanical stress can lead to the activation of MAPK signaling cascades, including ERKs, p38 kinases, and JNKs [40,41]. As shown in Figure 6a, we found the levels of p-ERK1/2, p-JNK, and p-p38 increased with the increase of hydrogel stiffness, suggesting that these MAPK signaling were activated in stiffer hydrogel. To explore the potential function of MAPK signaling in stiffer ECM-induced YAP activation, we treated HCC cells cultured on different stiffness of hydrogels with an MEK1/2 inhibitor (U0126), a JNK inhibitor (SP600125), or a p38 kinase inhibitor (SB203580). Western blot and immunofluorescent assay analysis showed both the JNK inhibitor and the p38 inhibitor, but not the MEK1/2 inhibitor, blocked stiffer ECM-induced YAP expression and its amount in the nucleus (Figure 6b–e). Then, qPCR were performed to exam the effects of the MAPKs inhibitors on the expression of YAP target genes. The results showed that the JNK inhibitor and the p38 inhibitor significantly inhibited stiffer matrix-induced upregulation of CYR61 and CTGF, suggesting that the JNK inhibitor and the p38 inhibitor inhibited the transcriptional activity of YAP (Figure 6f). The migration ability of HCC cells treated with JNK and p38 kinase inhibitors or knockdown of YAP was detected by using transwell assay, the result showed that these two inhibitors and knockdown of YAP all downregulated the migration ability of HCC cells on stiffer matrix. Moreover, these two inhibitors restored the migration ability of HCC cells cultured on stiffer matrix to the same level as that on soft matrix, which is consistent with the effect of YAP-siRNAs (Figure 6g). Together, these results demonstrated that the regulation of YAP activation by ECM stiffness is depending on JNK and p38 MAPK signaling cascades and ECM stiffness regulates the migration of HCC cells through MAPK-YAP mechanotransduction.

## 3. Discussion

**Figure 7:**
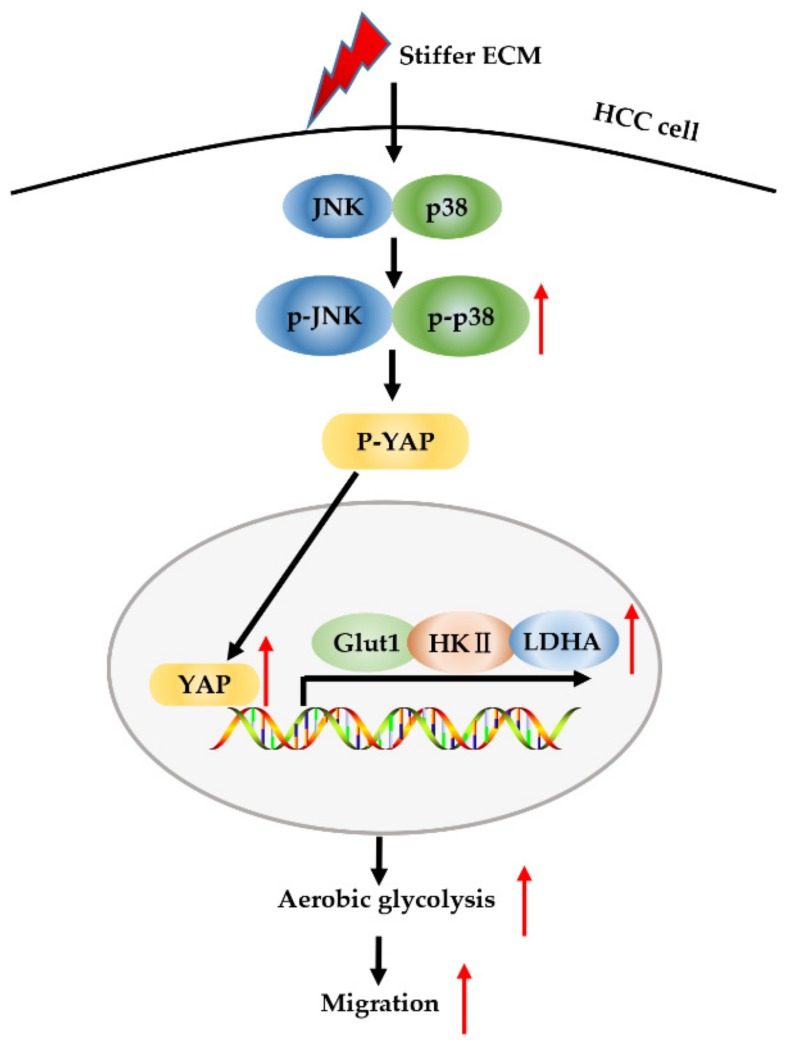
Schematic model for stiffer ECM-induced MAPK and YAP activation for metabolic reprogramming and migration of HCC cells. Stiffer ECM stimulates a signaling cascade through activation of MAPK and YAP to transduce biomechanical force. Stiffer ECM activates JNK and p38 and promotes their phosphorylation. The activated JNK and p38 dephosphorylate YAP and promote its localization in the nucleus. Then, activated YAP increases the expression of glycolysis-related genes, which in turn promotes cell glycolysis and migration.

Tumor niche ECM stiffening is known to promote tumor progression by influencing cancer cell behaviors and their sensitivity to therapeutics. Tumor cell metabolic reprogramming is another fundamental mediator of tumor progression, in addition to ECM stiffening [19]. YAP has been reported to serve not only as mechanosensors and mechanotransducers of mechanical cues [42] but also as an active mediator of cancer metabolic reprogramming [27], including aerobic glycolysis. In this study, we identified a MAPK-dependent regulatory network involving YAP that controls aerobic glycolysis during HCC cell migration caused by ECM stiffening (Figure 7).

The response of cells to mechanical force is a major determinant of cell behavior and is an energetically costly event [20]. Most studies investigating the effect of mechanical cues in tumorigenesis have focused on its cell behavior change capabilities, mechanical cues also play important roles in regulating cell metabolism. How mechanical force reprogramming cells metabolism by mechanotransduction to meet their needs for changing behavior remains poorly understood. Here, we investigated the mechanism underlying stiffer ECM-induced HCC cells migration. We observed that the inhibition of glycolysis remarkably suppressed the migration of HCC cells cultured on stiffer hydrogels. Additionally, we found that knockdown of YAP significantly inhibited the aerobic glycolysis of HCC cells cultured on stiffer hydrogels. These findings demonstrated that YAP functions as an important regulator to link enhanced migration capability and accelerated aerobic glycolysis of HCC cells induced by stiffer ECM.

In this study, we established that the JNK and p38 MAPK signaling serve as important regulators of YAP activation. The aberrant activation of YAP has been implicated in several human cancers, including in HCC, while the mutation frequency of the main core components of Hippo signaling pathway in HCC is low [23]. Therefore, the molecular mechanism leading to YAP activation has become the research focus. In this study, we found that treatment with a JNK inhibitor or a p38 inhibitor decreased YAP expression and transcriptional activity in HCC cells cultured on stiffer hydrogels. Previous studies have reported that MAPK pathway mediated mechanical-induced YAP activity, including tension [32] and shear stress [31]. Our findings indicated MAPKs pathway mediated YAP activity induced by another mechanical-ECM stiffness.

Hippo/YAP and MAPK signaling pathways have been recognized as important regulators of the initiation and progression of cancer [43,44,45]. Many studies have shown that YAP and MAPK act as mediators in regulating biological behaviors of cancer cells. In this study, we investigated the role of these pathways in stiffer ECM-induced HCC cells migration. The results showed that stiffer ECM increased the activity of YAP, JNK and p38. Interestingly, a JNK inhibitor or a p38 inhibitor decreased YAP expression and transcriptional activity. These results indicated that ECM stiffness acted as the upstream of the MAPK signaling axis, activated the phosphorylation of MAPK, then triggered YAP activation and the downstream cascade reaction. Therefore, we suggested that MAPK-YAP signaling cascade represent a possible and future mechanotransduction pathway to study, probably essential for promoting HCC cell migration processes.

Although we obtained some results, there are still some limitations in this study. First, it may be more convincing if these results can be further verified with in vivo experiments. Second, the regulation of ECM stiffness on the migration ability of liver cancer cells may also involve other pathways, so a more in-depth and systematic study will help us to reveal related mechanism more comprehensively. Our study provides a basis for exploring the mechanism of stiffer matrix promoting the occurrence and development of HCC, and further investigations still need to be carried out.

## 4. Materials and Methods

### 4.1. Preparation of Polyacrylamide Hydrogels

COL1-coated PA hydrogels with tunable mechanical properties were prepared by adjusting the relative concentrations of acrylamide and bis-acrylamide. PA hydrogels were prepared and their Young’s modulus was measured as we previously reported [46]. Briefly, 40% acrylamide and 2% bis-acrylamide to their desired concentrations mixed with 10% ammonium persulfate (APS; 1/200 volume) and N,N,N′,N′-Tetramethylethylenediamine (TEMED; 1/2000 volume). After polymerization, the gels were washed with distilled H_2_O thoroughly to remove unreacted reagents. Then the gels were further cross-linked using sulfosuccinimidyl 6-(4′-azido-2′-nitrophenylamino) hexanoate (sulfo-SANPAH; Thermo Fisher Scientific, MA, USA) under 365 nm ultraviolet exposure and conjugated with 0.1 mg/mL rat-tail COL1 (Hangzhou Shengyou Biotechnology, Zhejiang, China).

### 4.2. Cell Culture

The human HepG2 cell line was obtained from the American Type Culture Collection (ATCC, Manassas, WV, USA). The human MHCC97L cell line was obtained from the Liver Cancer Institute, Zhongshan Hospital, Fudan University (Shanghai, China). The human HCC cell lines HepG2 and MHCC97L were cultured in high-glucose Dulbecco’s modified Eagle’s medium (DMEM; Gbico, Thermo Fisher Scientific, MA, USA) supplemented of 10% fetal bovine serum (FBS; HyClone, UT, USA), 2 mM glutamine, penicillin (100 U/mL) and streptomycin (100 mg/mL).

### 4.3. Quantitative Real-Time PCR

Total RNA was extracted using RNA Extraction Kit (Takara, Kyoto, Japan). Reverse transcription was conducted by using PrimeScript™ RT reagent Kit (Perfect Real Time) (Takara, Kyoto, Japan) according to the manufacturer’s instructions. Real-time qPCR analyses were carried out with TB Green^®^ Premix Ex Taq™ II (Tli RNaseH Plus) (Takara, Kyoto, Japan). Expression levels are calculated relative to β-Actin. The sequences of primers were shown in Supplementary Table S1.

### 4.4. Western Blot Analysis

After washing with cold PBS, cells were lysed in RIPA supplemented with PMSF (Beyotime Biotechnology, Shanghai, China) protease inhibitor and phosphatase inhibitor (Beyotime Biotechnology, Shanghai, China). Lysates were adjusted to similar concentrations, mixed with 6× sample loading buffer and boiled for 5 min. The samples resolved by 4–20% SDS-PAGE gradient gels and transferred to polyvinylidene fluoride (PVDF) membranes (Millipore Corp, MA, USA). After blocked with 5% skimmed milk, the membranes were incubated with primary antibodies (YAP, p-YAP, Glut1, HKII, LDHA ERK1/2, p-ERK1/2, JNK, p-JNK, p38 and p-p38; Abcam, Cambridge, USA) (β-Actin; Zhongshan Golden Bridge Biotechnology, Beijing, China) overnight at 4 °C. Membranes were incubated with appropriate secondary antibodies (Zhongshan Golden Bridge Biotechnology, Beijing, China) for 1 h at room temperature, followed by detection using an ECL blotting analysis system (Bio-Rad, CA, USA).

### 4.5. Immunofluorescence Analysis

Cells were cultured on different stiffness of PA hydrogel in 24-well plates, after indicated treatments, cells were fixed in 4% paraformaldehyde for 15 min and washed three times with PBS. Next, cells were permeabilized treatment and blocked using 0.25% Triton X-100 and 0.5% BSA in PBS for 30 min, then washed three times with PBS. Then, cells were probed with primary anti-YAP antibodies (dilution, 1:300; Abcam, USA) diluted in 1% BSA/PBS overnight at 4 °C After five washes in PBS, cells were incubated with Alexa Fluor 488-labeled secondary antibodies (dilution, 1:500; Beyotime Biotechnology, Shanghai, China) diluted in 1% BSA/PBS for 1 h at room temperature. After five washes in PBS, nuclear was stained with 4′, 6′-diamidino-2-phenyl-indole dihydrochloride (DAPI; Solarbio, Beijing, China). After five washes in PBS, PA hydrogel were taken out from 24-well plate and placed on glass slide. Images were acquired using confocal microscope.

### 4.6. Cell Migration Assay

Transwell migration assay was conducted using transwell migration chambers (8 μm; Millipore, MA, USA). After indicated treatment, 5 × 10^4^ cells were suspended in 100 μL serum-free medium and placed in the upper chamber and the lower chamber was filled with 650 μL High-glucose DMEM with 10% FBS. After incubated for 12 h, the upper chamber was gently wiped with a cotton swab to remove the non-migrated cells, and the migrated cells on the underside of the chamber were stained with 0.1% crystal violet for 30 min. Each experiment was performed in triplicate, and the number of cells in three random fields on the underside of the filter was counted and averaged. The results were expressed as relative migrated cell number.

Wound scratch assay also used to detect cell migration. Cells were cultured for 48 h to reach 100% confluence in 6-well plate coated different stiffness of PA hydrogel. Straight wounds were created using a 200 µL sterile pipette tip. Two washes with media were performed after scratch to remove cell debris. Then, cells were cultured with serum-free culture medium. Wound healing was front photographed at indicated time point. Scratch areas were quantified using ImageJ software.

### 4.7. Quantification of Glucose Consumption and Lactate Production

Cells were seeded at 8 × 10^4^ cells per well on different stiffness of PA hydrogel in 12-well plate. 48 h later, the glucose and lactate levels in the medium were determined using the Glucose Assay Kit (Eton Bioscience, CA, USA) and Lactate Assay Kit II (Biovision, CA, USA) according to the manufacturer’s instructions, respectively. The glucose consumption and lactate production were then normalized to cell number.

### 4.8. siRNA Transfection

Specific siRNAs and negative control siRNA were purchased from Genepharma (Shanghai, China). The siRNA sequences were shown in Supplementary Table S2. For transfection experiments, siRNAs were transiently transfected into HCC cells using Lipofectamine 3000 (Invitrogen, Thermo Fisher Scientific, MA, USA) for 48 h, then cells were collected for further experiments.

### 4.9. Statistical Analysis

Data are expressed as means ± standard deviation (SD). *p*-values were analyzed by one-way analysis of variance (ANOVA) followed by Student’s t-tests. Statistical significances were accepted at *p* < 0.05.

## 5. Conclusions

In this study, we demonstrated that ECM stiffness-induced YAP activation contributes to metabolic reprogramming and migration of HCC cell, and MAPKs mediated the regulation of YAP expression and activation. Our results revealed the mechanism of HCC cell migration regulated by ECM mechanics and make us have better understand the influence of mechanical microenvironment on tumorigenesis and development.

## Supplementary Materials

The following are available online at https://www.mdpi.com/2072-6694/12/2/490/s1: Figure S1: Original western blots of Figure 2b. Figure S2: Original western blots of Figure 3a. Figure S3: Original western blots of Figure 4a. Figure S4: Original western blots of Figure 5a. Figure S5: Original western blots of Figure 5c. Figure S6: Original western blots of Figure 6a. Figure S7: Original western blots of Figure 6b. Figure S8: Original western blots of Figure 6c. Figure S9: Original western blots of Figure 6d. Table S1: The list of primers. Table S2: The list of siRNAs.

## References

[1] Yu H., Mouw J.K., Weaver V.M. (2011). Forcing form and function: Biomechanical regulation of tumor evolution. Trends Cell Biol..

[2] Wirtz D., Konstantopoulos K., Searson P.C. (2011). The physics of cancer: The role of physical interactions and mechanical forces in metastasis. Nature.

[3] Nagelkerke A., Bussink J., Rowan A.E., Span P.N. (2015). The mechanical microenvironment in cancer: How physics affects tumours. Semin. Cancer Biol..

[4] Bissell M.J., Hines W.C. (2011). Why don’t we get more cancer? A proposed role of the microenvironment in restraining cancer progression. Nat. Med..

[5] Acerbi I., Cassereau L., Dean I., Shi Q., Au A., Park C., Chen Y.Y., Liphardt J., Hwang E.S., Weaver V.M. (2015). Human breast cancer invasion and aggression correlates with ECM stiffening and immune cell infiltration. Integr. Biol. (Camb.).

[6] Dong Y., Xie X., Wang Z., Hu C., Zheng Q., Wang Y., Chen R., Xue T., Chen J., Gao D. (2014). Increasing matrix stiffness upregulates vascular endothelial growth factor expression in hepatocellular carcinoma cells mediated by integrin beta1. Biochem. Biophys. Res. Commun..

[7] Pang M., Teng Y., Huang J., Yuan Y., Lin F., Xiong C. (2017). Substrate stiffness promotes latent TGF-beta1 activation in hepatocellular carcinoma. Biochem. Biophys. Res. Commun..

[8] Gnoni A., Santini D., Scartozzi M., Russo A., Licchetta A., Palmieri V., Lupo L., Faloppi L., Palasciano G., Memeo V. (2015). Hepatocellular carcinoma treatment over sorafenib: epigenetics, microRNAs and microenvironment. Is there a light at the end of the tunnel?. Expert Opin. Ther. Targets.

[9] Zhao G., Cui J., Qin Q., Zhang J., Liu L., Deng S., Wu C., Yang M., Li S., Wang C. (2010). Mechanical stiffness of liver tissues in relation to integrin beta1 expression may influence the development of hepatic cirrhosis and hepatocellular carcinoma. J. Surg. Oncol..

[10] Schrader J., Gordon-Walker T.T., Aucott R.L., van Deemter M., Quaas A., Walsh S., Benten D., Forbes S.J., Wells R.G., Iredale J.P. (2011). Matrix stiffness modulates proliferation, chemotherapeutic response, and dormancy in hepatocellular carcinoma cells. Hepatology.

[11] Kim S.M., Yun M.R., Hong Y.K., Solca F., Kim J.-H., Kim H.-J., Cho B.C. (2013). Glycolysis inhibition sensitizes non-small cell lung cancer with T790M mutation to irreversible EGFR inhibitors via translational suppression of Mcl-1 by AMPK activation. Mol. Cancer Ther..

[12] Ooi A.T., Gomperts B.N. (2015). Molecular pathways: Targeting cellular energy metabolism in cancer via inhibition of SLC2A1 and LDHA. Clin. Cancer Res..

[13] Gill K.S., Fernandes P., O’Donovan T.R., McKenna S.L., Doddakula K.K., Power D.G., Soden D.M., Forde P.F. (2016). Glycolysis inhibition as a cancer treatment and its role in an anti-tumour immune response. Biochim. Biophys. Acta Rev. Cancer.

[14] Cairns R.A., Harris I.S., Mak T.W. (2011). Regulation of cancer cell metabolism. Nat. Rev. Cancer.

[15] Huang Q., Tan Y., Yin P., Ye G., Gao P., Lu X., Wang H., Xu G. (2013). Metabolic characterization of hepatocellular carcinoma using nontargeted tissue metabolomics. Cancer Res..

[16] Iansante V., Choy P.M., Fung S.W., Liu Y., Chai J.-G., Dyson J., Del Rio A., D’Santos C., Williams R., Chokshi S. (2015). PARP14 promotes the Warburg effect in hepatocellular carcinoma by inhibiting JNK1-dependent PKM2 phosphorylation and activation. Nat. Commun..

[17] Kitamura K., Hatano E., Higashi T., Narita M., Seo S., Nakamoto Y., Yamanaka K., Nagata H., Taura K., Yasuchika K. (2011). Proliferative activity in hepatocellular carcinoma is closely correlated with glucose metabolism but not angiogenesis. J. Hepatol..

[18] Amann T., Maegdefrau U., Hartmann A., Agaimy A., Marienhagen J., Weiss T.S., Stoeltzing O., Warnecke C., Schölmerich J., Oefner P.J. (2009). GLUT1 expression is increased in hepatocellular carcinoma and promotes tumorigenesis. Am. J. Pathol..

[19] Bertero T., Gaggioli C. (2019). Mechanical forces rewire metabolism in the tumor niche. Mol. Cell. Oncol..

[20] Bays J.L., Campbell H.K., Heidema C., Sebbagh M., Demali A., Roy J., City I., Biology C., Roy J., Lucille A. (2017). Linking E-cadherin mechanotransduction to cell metabolism through force-mediated activation of AMPK. Nat. Cell Biol..

[21] Bertero T., Oldham W.M., Cottrill K.A., Pisano S., Vanderpool R.R., Yu Q., Zhao J., Tai Y., Tang Y., Zhang Y.-Y. (2016). Vascular stiffness mechanoactivates YAP/TAZ-dependent glutaminolysis to drive pulmonary hypertension. J. Clin. Invest..

[22] Dufort C.C., Paszek M.J., Weaver V.M. (2011). Balancing forces: architectural control of mechanotransduction. Nat. Rev. Mol. Cell Biol..

[23] Zhang S., Zhou D. (2019). Role of the transcriptional coactivators YAP/TAZ in liver cancer. Curr. Opin. Cell Biol..

[24] Du K., Hyun J., Premont R.T., Choi S.S., Michelotti G.A., Swiderska-Syn M., Dalton G.D., Thelen E., Rizi B.S., Jung Y. (2018). Hedgehog–YAP signaling pathway regulates glutaminolysis to control hepatic stellate cell activation. Gastroenterology.

[25] Cosset É., Ilmj?rv S., Dutoit V., Elliott K., von Schalscha T., Camargo M.F., Reiss A., Moroishi T., Seguin L., Gomez G. (2017). Glut3 addiction is a druggable vulnerability for a molecularly defined subpopulation of glioblastoma. Cancer Cell.

[26] Plouffe S., Meng Z., Lin K., Lin B., Hong A., Chun J., Guan K.-L. (2016). Characterization of Hippo pathway components by gene inactivation. Mol. Cell.

[27] Zhang X., Zhao H., Li Y., Xia D., Yang L., Ma Y., Li H. (2018). The role of YAP/TAZ activity in cancer metabolic reprogramming. Mol. Cancer.

[28] Kim W., Khan S.K., Gvozdenovic-Jeremic J., Kim Y., Dahlman J., Kim H., Park O., Ishitani T., Jho E., Gao B. (2017). Hippo signaling interactions with Wnt/β-catenin and Notch signaling repress liver tumorigenesis. J. Clin. Invest..

[29] Jeong S.-H., Kim H.-B., Kim M.-C., Lee J., Lee J.H., Kim J.-H., Kim J.-W., Park W.-Y., Kim S.-Y., Kim J.B. (2018). Hippo-mediated suppression of IRS2/AKT signaling prevents hepatic steatosis and liver cancer. J. Clin. Invest..

[30] Hoffman L., Jensen C.C., Yoshigi M., Beckerle M. (2017). Mechanical signals activate p38 MAPK pathway-dependent reinforcement of actin via mechanosensitive HspB1. Mol. Biol. Cell.

[31] Qin X., Li J., Sun J., Liu L., Chen D., Liu Y. (2019). Low shear stress induces ERK nuclear localization and YAP activation to control the proliferation of breast cancer cells. Biochem. Biophys. Res. Commun..

[32] Liu Z., Wu H., Jiang K., Wang Y., Zhang W., Chu Q., Li J., Huang H., Cai T., Ji H. (2016). MAPK-mediated YAP activation controls mechanical-tension-induced pulmonary alveolar regeneration. Cell Rep..

[33] Wong V.W.S., Vergniol J., Wong G.L.H., Foucher J., Chan H.L.Y., Le Bail B., Choi P.C.L., Kowo M., Chan A.W.H., Merrouche W. (2010). Diagnosis of fibrosis and cirrhosis using liver stiffness measurement in nonalcoholic fatty liver disease. Hepatology.

[34] Swift J., Ivanovska I.L., Buxboim A., Harada T., Dingal P.C.D.P., Pinter J., Pajerowski J.D., Spinler K.R., Shin J.-W., Tewari M. (2013). Nuclear lamin-A scales with tissue stiffness and enhances matrix-directed differentiation. Science.

[35] Masuzaki R., Tateishi R., Yoshida H.H., Sato T., Ohki T., Goto T., Yoshida H.H., Sato S., Sugioka Y., Ikeda H. (2007). Assessing liver tumor stiffness by transient elastography. Hepatol. Int..

[36] Bensinger S.J., Christofk H.R. (2012). New aspects of the Warburg effect in cancer cell biology. Semin. Cell Dev. Biol..

[37] Chen R., Zhu S., Fan X.G., Wang H., Lotze M.T., Rd Z.H., Billiar T.R., Kang R., Tang D. (2017). HMGB1 controls liver cancer initiation through YAP-dependent aerobic glycolysis. PLoS ONE.

[38] Hay N. (2016). Reprogramming glucose metabolism in cancer: Can it be exploited for cancer therapy?. Nat. Rev. Cancer.

[39] Zhao B., Wei X., Li W., Udan R.S., Yang Q., Kim J., Xie J., Ikenoue T., Yu J., Li L. (2007). Inactivation of YAP oncoprotein by the Hippo pathway is involved in cell contact inhibition and tissue growth control. Genes Dev..

[40] Kong D., Zheng T., Zhang M., Wang D., Du S., Li X., Fang J., Cao X. (2013). Static mechanical stress induces apoptosis in rat endplate chondrocytes through MAPK and mitochondria-dependent caspase activation signaling pathways. PLoS ONE.

[41] 41. Pereira A.M., Tudor C., Pouille P.A., Shekhar S., Kanger J.S., Subramaniam V., Martín-Blanco E. (2014). Plasticity of the MAPK signaling network in response to mechanical stress. PLoS ONE.

[42] Low B.C., Pan C.Q., Shivashankar G.V., Bershadsky A., Sudol M., Sheetz M. (2014). YAP/TAZ as mechanosensors and mechanotransducers in regulating organ size and tumor growth. FEBS Lett..

[43] Panciera T., Azzolin L., Cordenonsi M., Piccolo S. (2017). Mechanobiology of YAP and TAZ in physiology and disease. Nat. Rev. Mol. Cell Biol..

[44] Zanconato F., Cordenonsi M., Piccolo S. (2016). YAP/TAZ at the Roots of Cancer. Cancer Cell.

[45] Wagner E.F., Nebreda A.R. (2009). Signal integration by JNK and p38 MAPK pathways in cancer development. Nat. Rev. Cancer.

[46] Tian B., Luo Q., Ju Y., Song G. (2019). A soft matrix enhances the cancer stem cell phenotype of HCC cells. Int. J. Mol. Sci..

