# Supplementary material for: Stiffer Matrix Accelerates Migration of Hepatocellular Carcinoma Cells through Enhanced Aerobic Glycolysis Via the MAPK-YAP Signaling
Source: Cancers (Basel). 2020 Feb 19;12(2):490. doi: 10.3390/cancers12020490 (PMC7072284; doi:10.3390/cancers12020490)
Supplement: Supplementary file 1 [file cancers-12-00490-s001.pdf]

cancers

Article

Stiffer Matrix Accelerates Migration of Hepatocellular Carcinoma Cells through Enhanced Aerobic Glycolysis Via the MAPK-YAP Signaling

Qiu-Ping Liu [1], Qing Luo [1], Bin Deng [1], Yang Ju [2] and Guan-Bin Song [1,*]

[1] Key Laboratory of Biorheological Science and Technology, Ministry of Education, College of Bioengineering, Chongqing University, Chongqing 400030, China; liuqp@cqu.edu.cn (Q.-P.L); qing.luo@cqu.edu.cn (Q.L.); dengbin2018@cqu.edu.cn (B.D.)

[2] Department of Mechanical Science and Engineering, Nagoya University, Nagoya 464-8603, Japan; ju@mech.nagoya-u.ac.jp

* Correspondence: song9973@163.com; Tel./Fax: +86-23-65102507

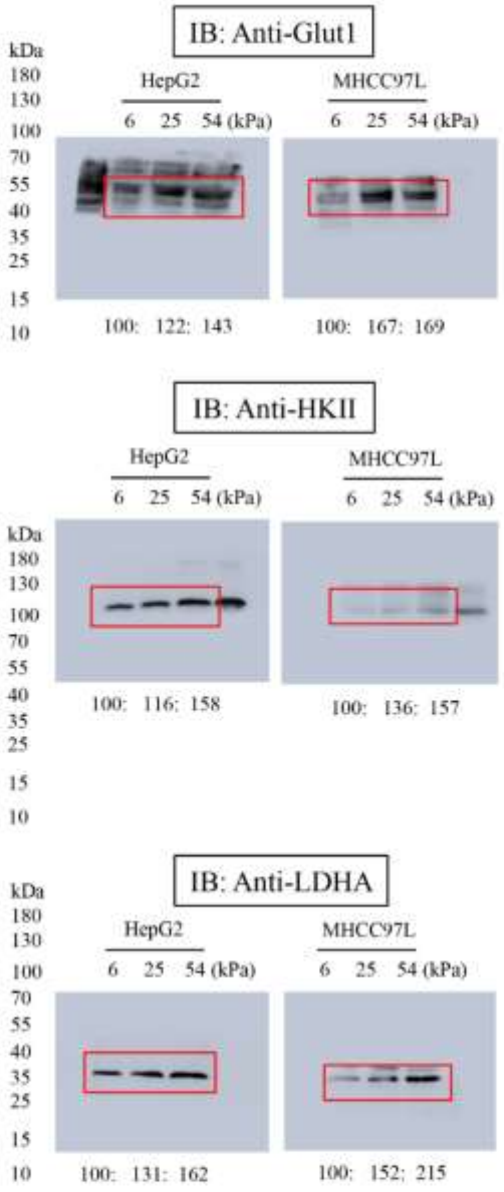

Figure S1: Original western blots of Figure 2b.

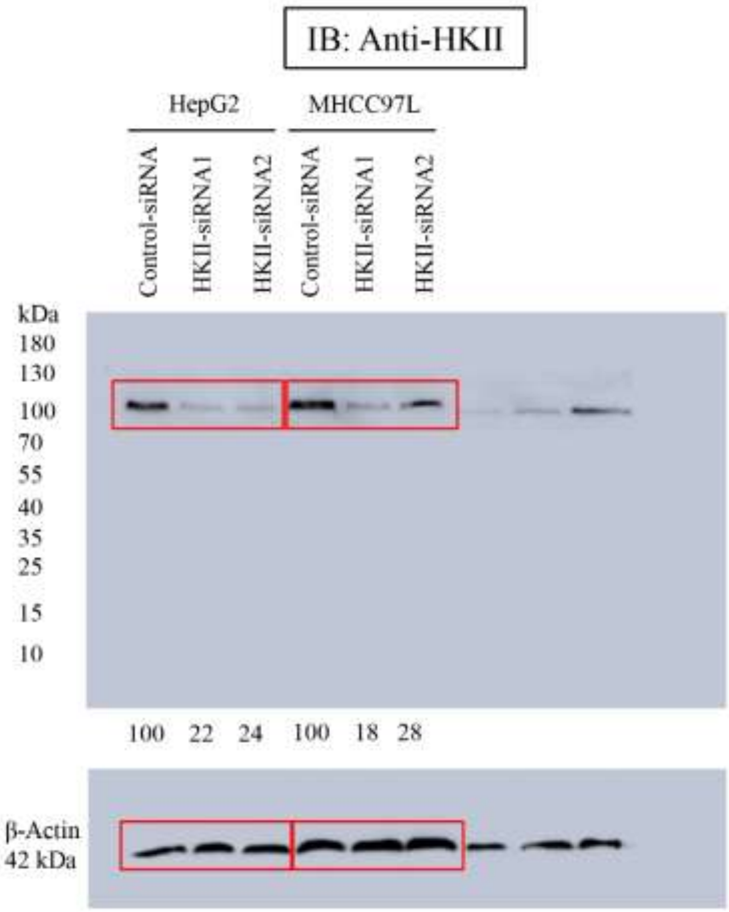

Figure S2: Original western blots of Figure 3a.

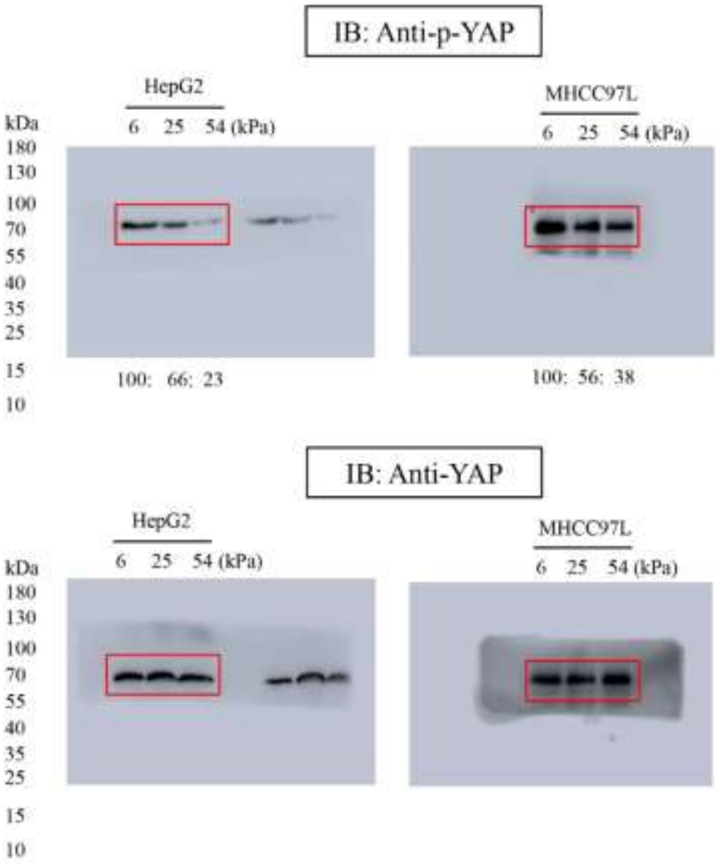

Figure S3: Original western blots of Figure 4a.

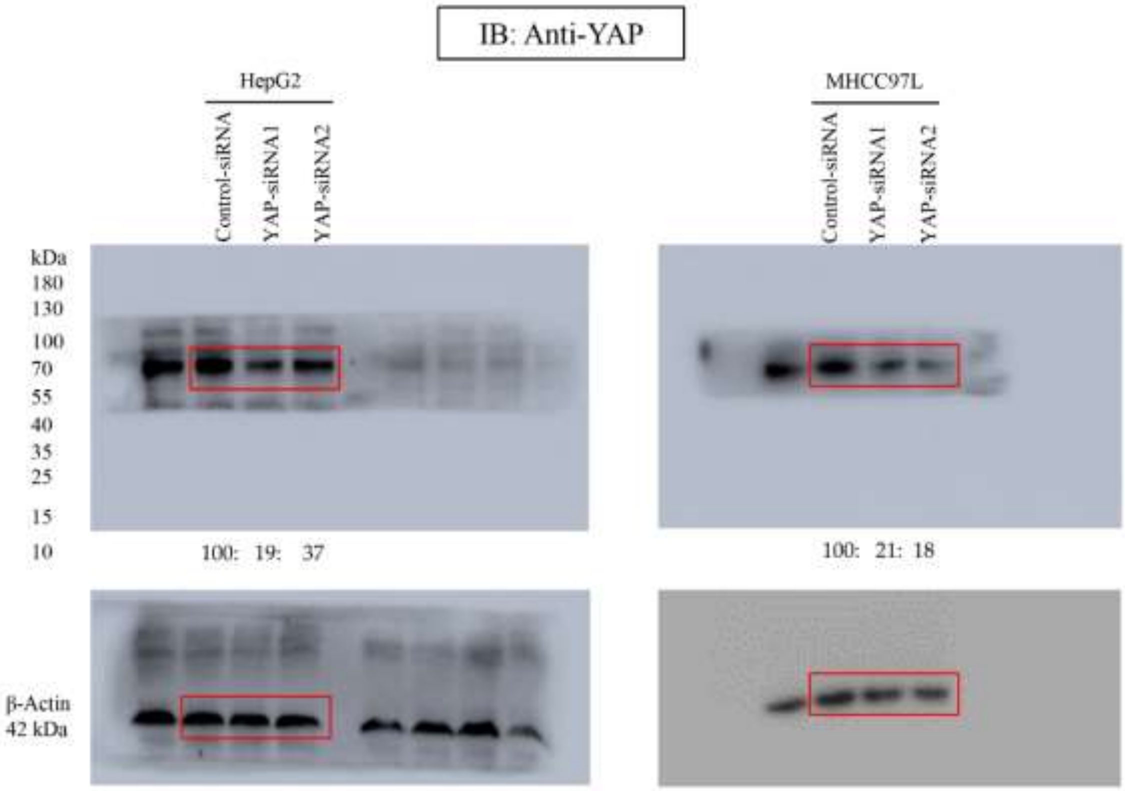

Figure S4: Original western blots of Figure 5a.

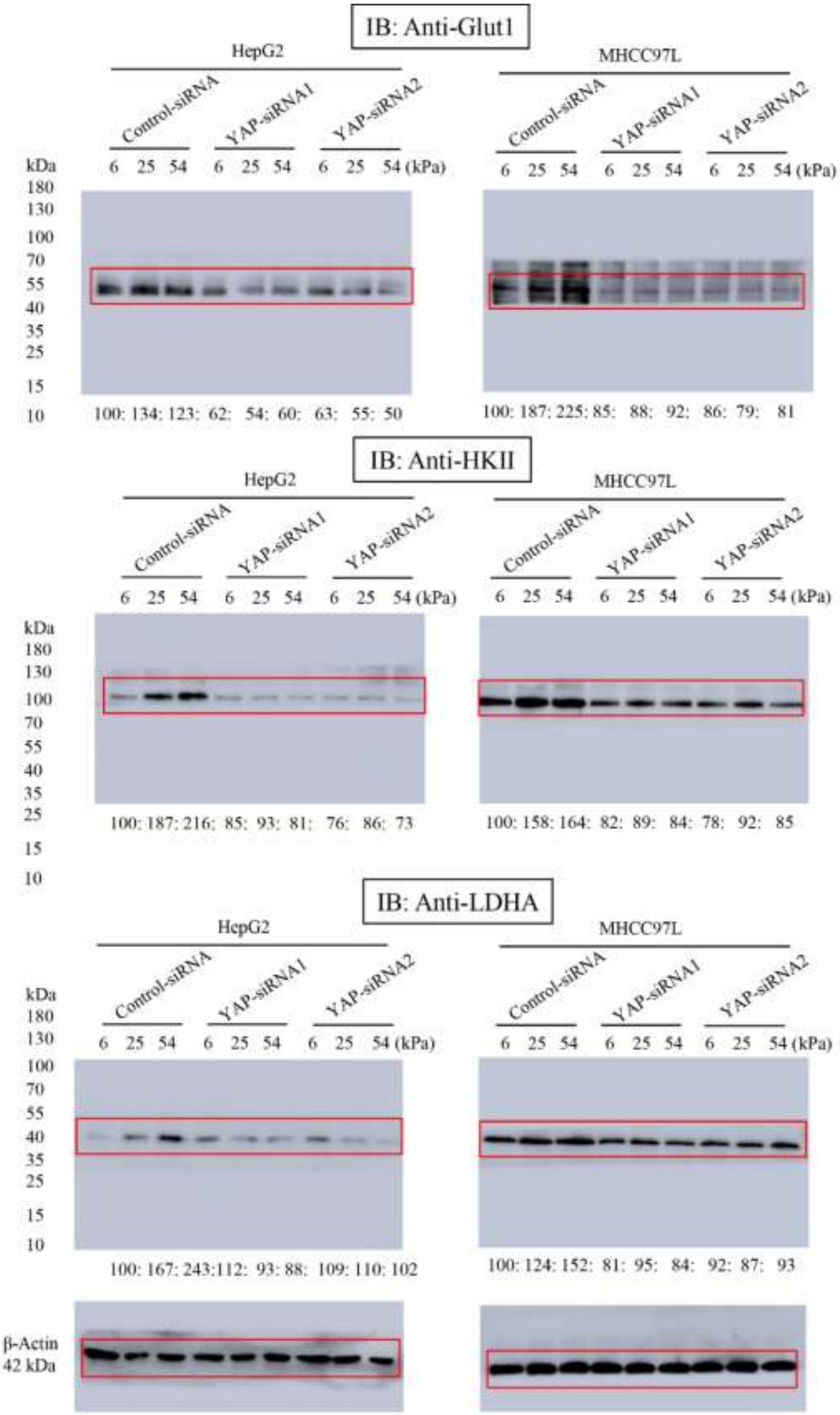

Figure S5: Original western blots of Figure 5c.

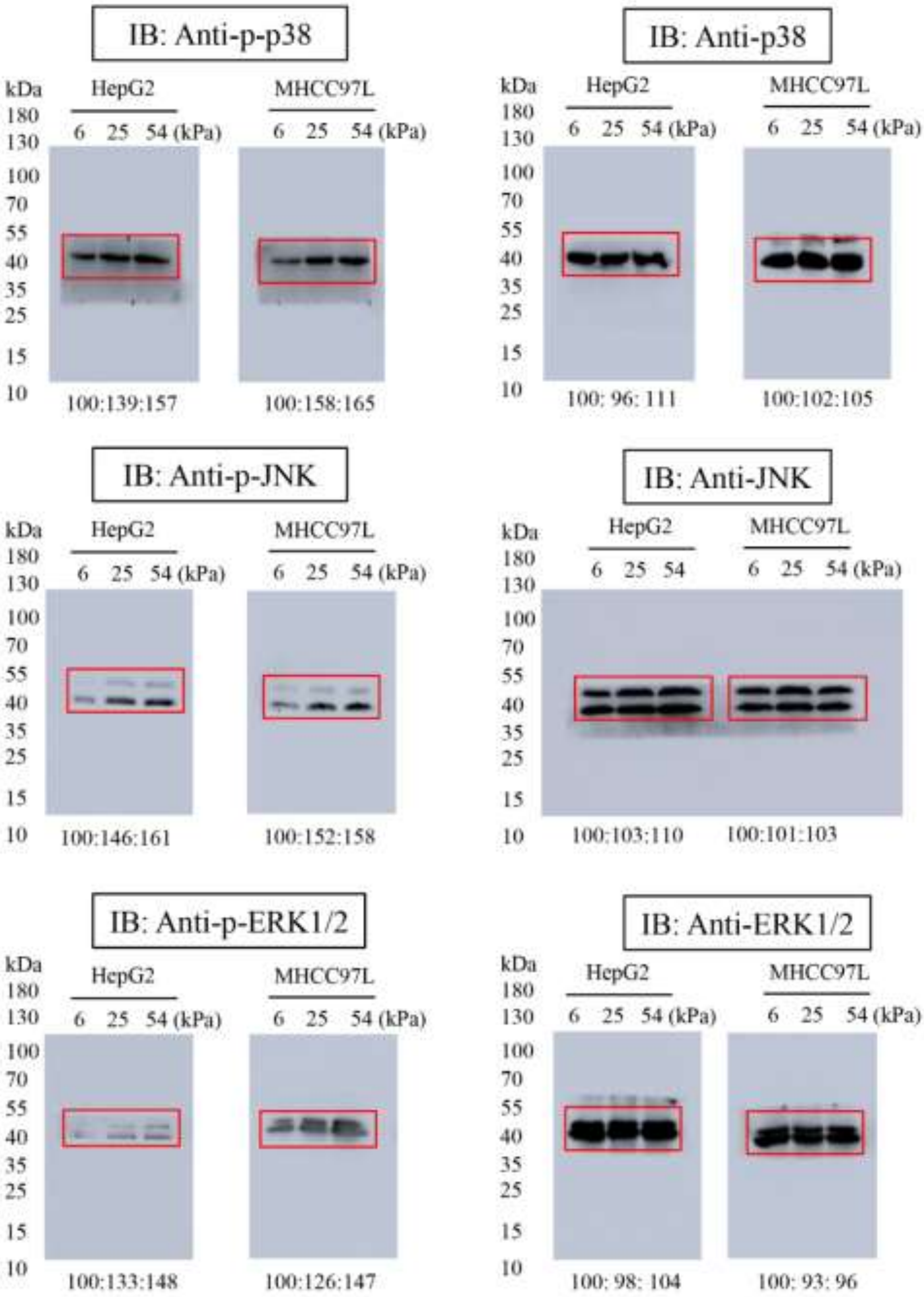

Figure S6: Original western blots of Figure 6a.

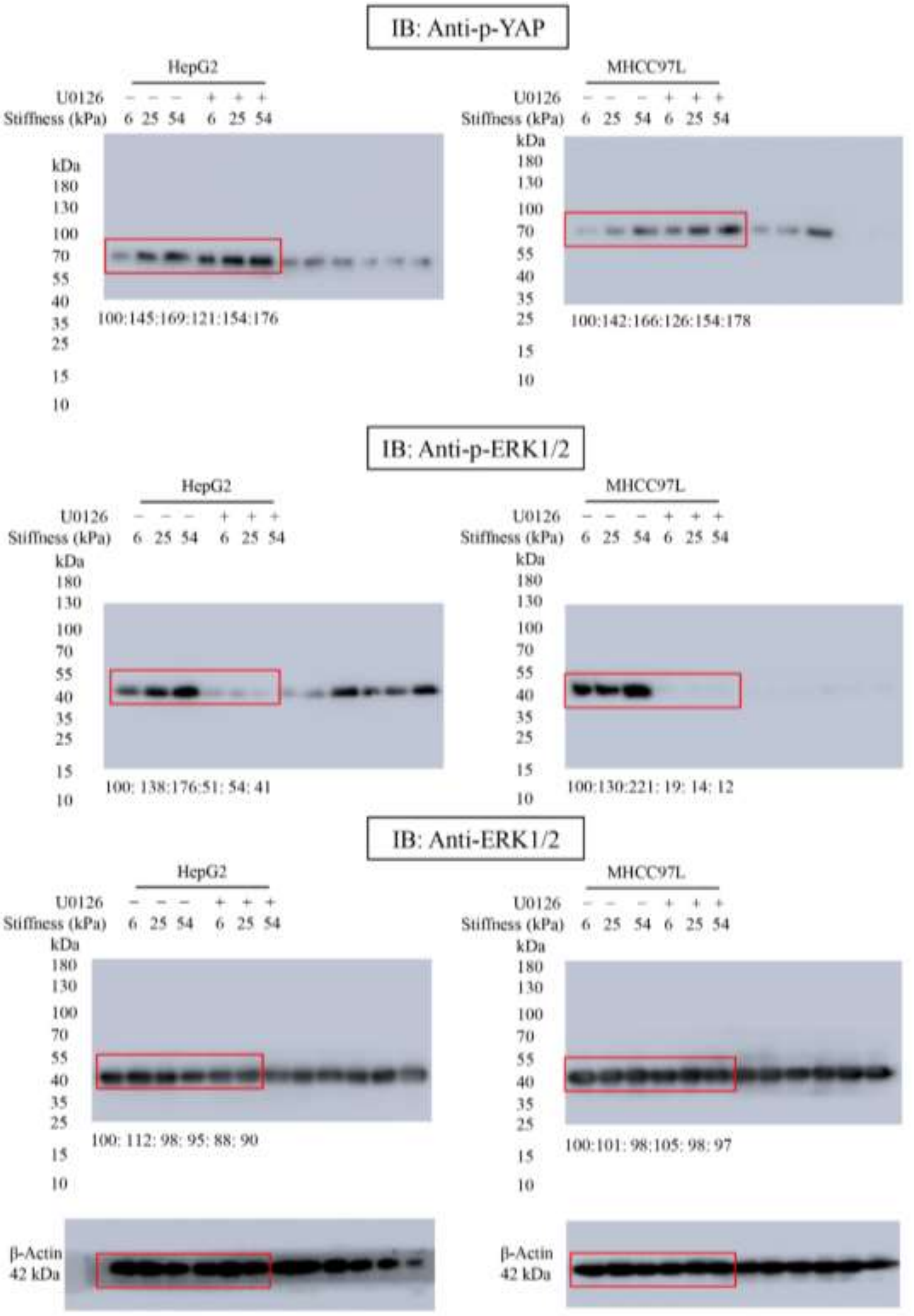

Figure S7: Original western blots of Figure 6b.

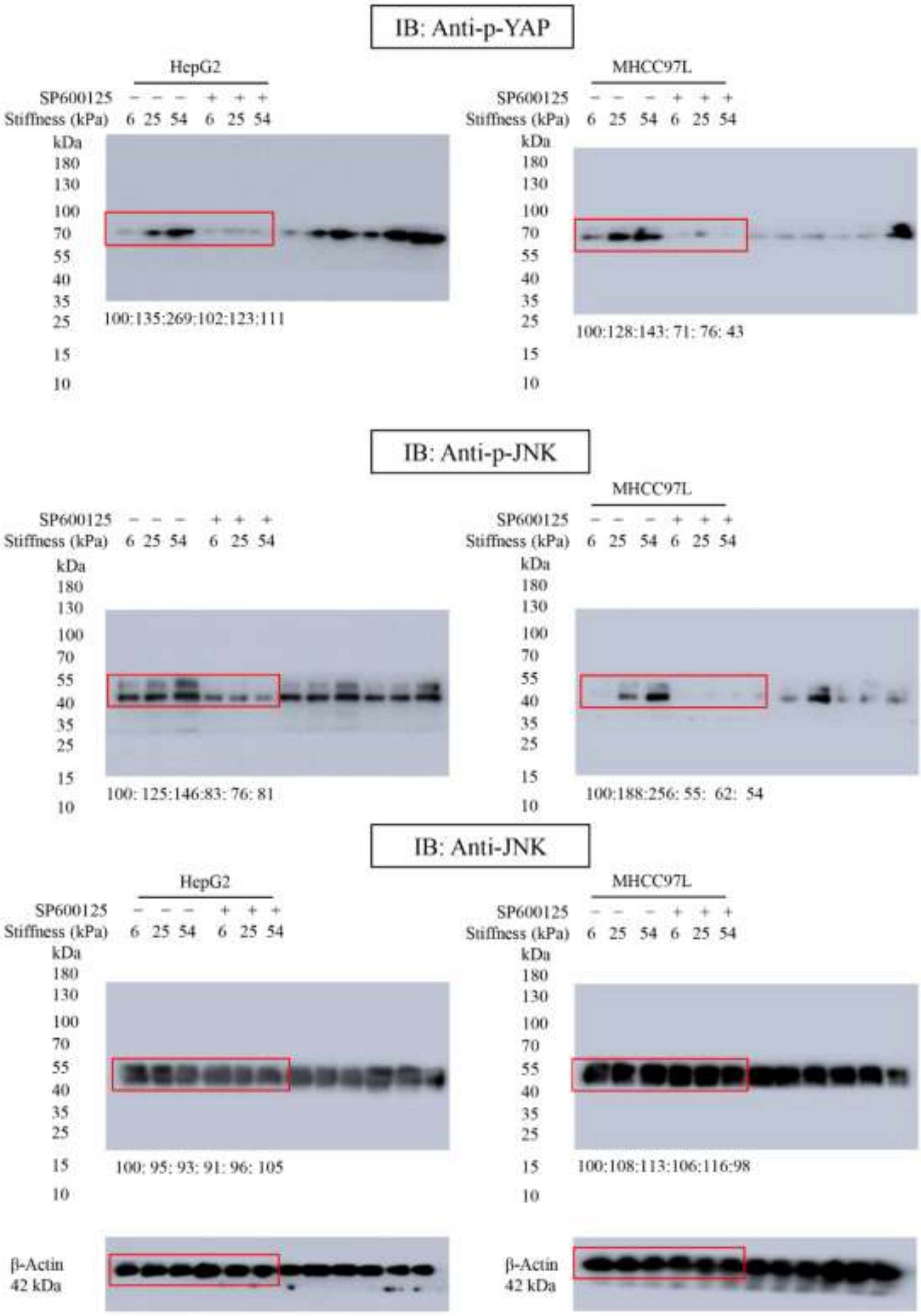

Figure S8: Original western blots of Figure 6c.

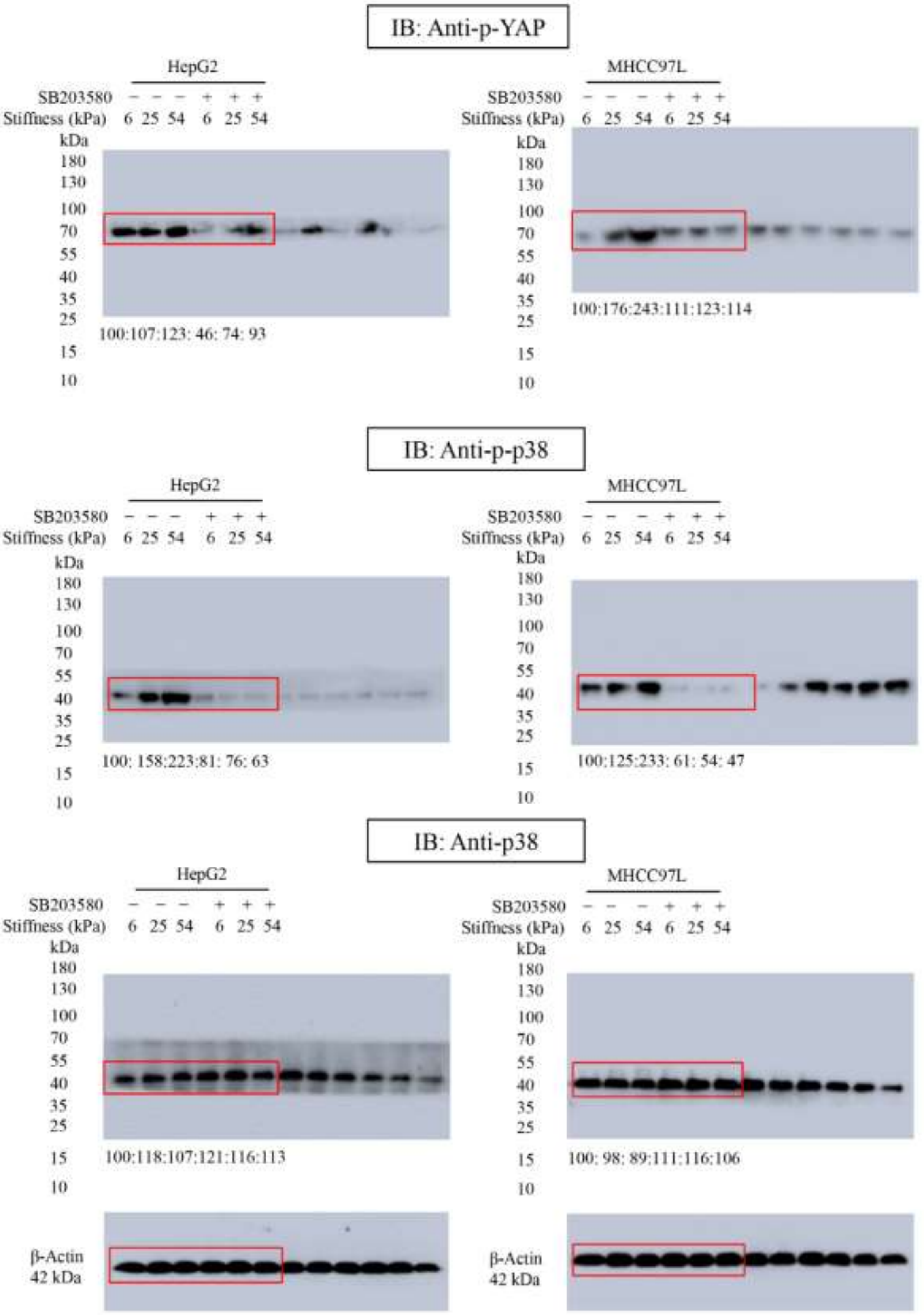

Figure S9: Original western blots of Figure 6d.

Table 1. The list of primers.

Target genes	Name	Primer sequences (5´-3´)
Glut1	Forward	AACTCTTCAGCCAGGGTCCAC
	Reverse	CACAGTGAAGATGATGAAGAC
HK2	Forward	TGGAGGGACCAACTTCCGTGTGCT
	Reverse	TCAAACAGCTGGGTGCCACTGC
LDHA	Forward	ATGGCAACTCTAAAGGATCAGC
	Reverse	CCAACCCCAACAACTGTAATCT
CYR61	Forward	ACCGCTCTGAAGGGGATCT
	Reverse	ACTGATGTTTACAGTTGGGCTG
CTGF	Forward	AGGAGTGGGTGTGTGACGA
	Reverse	CCAGGCAGTTGGCTCTAATC
β-Actin	Forward	AAAGACCTGTACGCCAACAC
	Reverse	GTCATACTCCTGCTTGCTGAT

Table 2. The list of siRNAs.

Target genes	Name	Sequences (5´-3´)
YAP	YAP-siRNA1 sense	GACAUCUUCUGGUCAGAGA
	YAP-siRNA1 antisense	UCUCUGACCAGAAGAUGUC
	YAP-siRNA2 sense	CUGGUCAGAGAUACUUCUU
	YAP-siRNA2 antisense	AAGAAGUAUCUCUGACCAG
HKⅡ	HKⅡ-siRNA1 sense	CACGAUGAAAUUGAACCUGGU
	HKⅡ-siRNA1 antisense	ACCAGGUUCAAUUUCAUCGUG
	HKⅡ-siRNA2 sense	CCUGGGUGAGAUUGUCCGUAA
	HKⅡ-siRNA2 antisense	UUACGGACAAUCUCACCCAGG
